# A Comparative Investigation of Aerosol Generation and Exposure Risk During Access Cavity Preparation With or Without Rubber Dam Application

**DOI:** 10.7759/cureus.61758

**Published:** 2024-06-05

**Authors:** Anugeet K Dev, Aakash Gupta, Sasmita Dalai

**Affiliations:** 1 Conservative Dentistry and Endodontics, Baba Jaswant Singh Dental College, Hospital and Research Institute, Ludhiana, IND; 2 Dentistry, All India Institute of Medical Sciences, Bathinda, Bathinda, IND

**Keywords:** endodontic access cavity, settle plates, respiratory infections, rubber dam, aerosol

## Abstract

Background: Aerosols generated during dental procedures have taken the forefront of discussion in dentistry. Due to the nature of their work, dental professionals face a significant risk of exposure to various biological hazards, such as saliva, blood, aerosols, and droplets. Aerosols, which are tiny particles with a diameter of less than 50µm, have a unique property that allows them to stay suspended in the air for extended periods. This is primarily due to their small size and lightweight nature which makes them highly susceptible to air currents and prevents them from quickly settling down. As a result, these aerosols can linger in the atmosphere, creating a potential risk for respiratory infections.

Aim: The aim is to evaluate and compare the efficacy of rubber dams in preventing aerosols generated cross-contamination.

Methods and material: This in-vivo experimental study comprised 60 individuals who were suggested for root canal treatment in the mandibular first permanent molar tooth. The passive air sampling technique using “settle plates” was applied to investigate microbial fallout during access opening with and without rubber dam application. Sheep blood agar plates were used to do the colony forming unit (CFU) count. All patients were randomly divided into two groups comprising 30 patients each based on usage of rubber dam application or not, i.e., Group I: Without rubber dam application and Group II: With rubber dam application.

Results: Using a rubber dam while performing an endodontic procedure significantly impacts decreased aerosol generation at 0.5-m and 2-m distances than its counterpart with a p-value < 0.01.

Conclusion: Using a rubber dam during endodontic procedures reduces the likelihood of aerosol generation, thereby decreasing the risk of cross-contamination and lowering the susceptibility of dental professionals to respiratory illnesses.

## Introduction

Given the potential for human-to-human microbial transmission, disease prevention inside the healthcare system is a noble technique that has gained importance, particularly in light of the introduction of increasingly pathogenic microbial variations [1]. It is rightly said, “Prevention is better than cure.” Aerosols generated during dental procedures are the prime source of potential hazards [2]. The potential hazard of aerosols lies in their ability to carry harmful substances or pathogens. For example, aerosols can contain infectious agents such as bacteria or viruses which can cause respiratory infections like the flu or pneumonia [3].

For instance, in crowded indoor spaces or healthcare settings with limited ventilation, aerosols produced during dental processes have the potential to spread through the air conditioning system, which can help spread pathogenic microbes to distant locations [4]. Given the ongoing alarming situation, it is imperative to effectively manage the spread of infections caused by airborne transmission of biological pathogens. So, this study aims to check the efficacy of rubber dam application in reinforcing more stringent biosafety measures within dental facilities.

## Materials and methods

Subjects and methods

A sum of 60 patients undergoing root canal therapy in the mandibular first permanent molar was selected for this study. Patients were informed about the study and written informed consent was signed from the patient.

Preparation of participants

Sixty patients who were diagnosed with symptomatic irreversible pulpitis in the mandibular first permanent molar based on radiographical and clinical examination were informed about the procedure. 2%lignocaine with 1:200,000 adrenaline was used for giving inferior alveolar nerve block. Thirty patients were randomly selected based on the flipping coin method for without rubber dam application while doing the procedure (Group I) and 30 patients were randomly chosen based on the flipping coin method for rubber dam application during the procedure (Group II). Access cavity preparation was done in both groups with new autoclaved Endo Access bur number EA10 (MANI, Inc., Japan) followed by refinement with new Endo Z bur (Dentsply, Tulsa, Oklahoma) in each case. Distilled water was used while doing the procedure and all sanitary pipes and dental chair pipes were cleaned before the procedure with Durr Dental solution to avoid any false results.

Microbial collection

The passive air sampling technique using “settle plates” was applied to investigate microbial fallout during access opening with a rubber dam and without rubber dam application. Sheep blood agar plates were used to do the colony forming unit (CFU) count.

Microbial fallout samples were collected from a total of 60 access cavity preparations including 30 without rubber dam application (Group I) and 30 with rubber dam application (Group II). The agar plates were exposed to the air in the operatory for 30 min.

All samples were collected in the endodontic resident’s operatory with closed doors. The high-efficiency particulate air (HEPA) filter was left on for 30 minutes before starting the procedure. The sample size was determined using the formula.

n = (Zα/2)^2^ s^2^/d^2^, where n is for minimum sample size, Zα is critical value from the standard normal distribution corresponding to the desired confidence level, s is the standard deviation, and d is the desired margin of error.

Placement of settle plates

**Figure 1:**
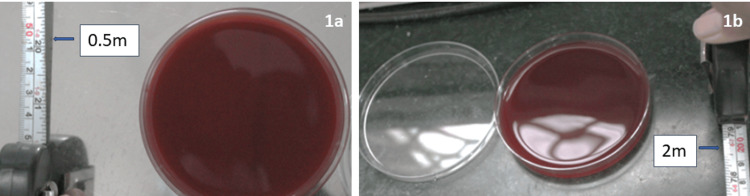
Plates placed at 0.5 m (a) and 2 m (b) distance from patient’s mouth.

The plates were spatially distributed at 1-m high from the floor at two different sites (Figures 1a, 1b): one is 0.5 m directly in front of the patient’s mouth and other is 2 m directly in front of the patient’s mouth.

**Figure 2:**
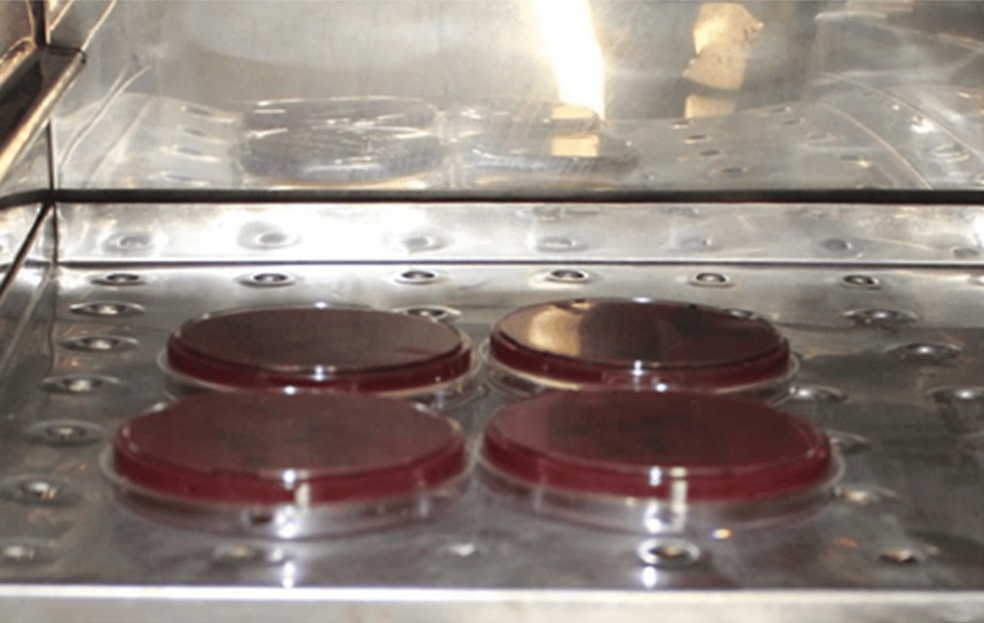
The agar plates were incubated at 37º C in aerobic conditions for 48 hours.

After collecting the samples, the agar plates were incubated at 37º C in aerobic conditions for 48 hours (Figure 2).

**Figure 3:**
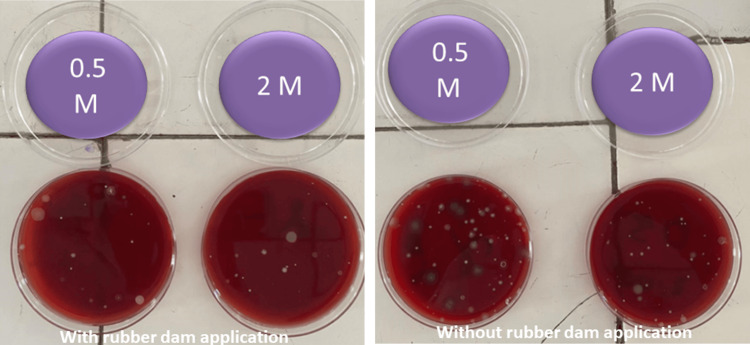
CFU count at different locations with (left) or without rubber dam application (right). CFU - colony forming unit

Following this, CFU count was assessed for both the groups at different sites (Figure 3) and values were calculated and compared.

Clinical significance

Microbial contamination occurs mainly during aerosol-generating dental procedures which can be prevented to a greater extent by the judicious use of rubber dams while performing endodontic procedures and preventing dental health professionals from various respiratory infections.

Statistical analysis

The information for the current investigation was entered into Microsoft Excel 2007 and processed using SPSS statistical software version 23.0 (IBM Corp., Armonk, NY). The descriptive statistics involved mean, standard deviation, frequency, and percentage. The significance level for this study was established at 5%.

The independent t-tests will be employed for conducting the intergroup comparison, while the paired t-test will be utilized for the intragroup comparison. To examine the distribution of the data, the Shapiro-Wilk test was employed, and the homogeneity of the variables was explored using Levene's test.

## Results

**Figure 4:**
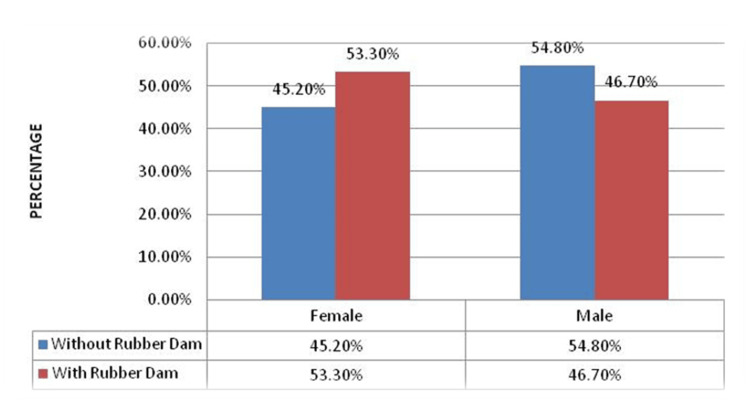
Graph showing gender distribution of study subjects.

Based on the gender distribution, 45.2% (n=13) of the subjects in the without rubber dam group were females and 54.8% (n=17) were males. In the subjects with a rubber dam, 53.3% (n=16) were females and 46.7% (n=14) were males (Figure 4).

The mean CFU count at the 0.5 m distance was 21.741±2.081 in the without rubber dam group and 17.400±1.734 in the rubber dam group (Table 1). The mean CFU count was significantly lower in the rubber dam group as compared to the without rubber dam group.

**Table 1:** Aerosol concentration at 0.5 m distance.

Groups	Mean	Std. Deviation	Std. Error Mean	P-value	Significance (p < 0.001)
Without Rubber Dam	21.741	2.081	0.373	0.001	Significant
With Rubber Dam	17.400	1.734	0.316		

The mean CFU count at the 2 m distance was 10.516±1.338 in the without rubber dam group and 9.433±1.612 in the rubber dam group (Table 2). The mean CFU count was significantly lower in the rubber dam group as compared to the without rubber dam group.

**Table 2:** Aerosol concentration at 2 m distance.

Groups	Mean	Std. Deviation	Std. Error Mean	P-value	Significance (p < 0.01)
Without Rubber Dam	10.516	1.338	0.240	0.006	Significant
With Rubber Dam	9.433	1.612	0.294		

**Figure 5:**
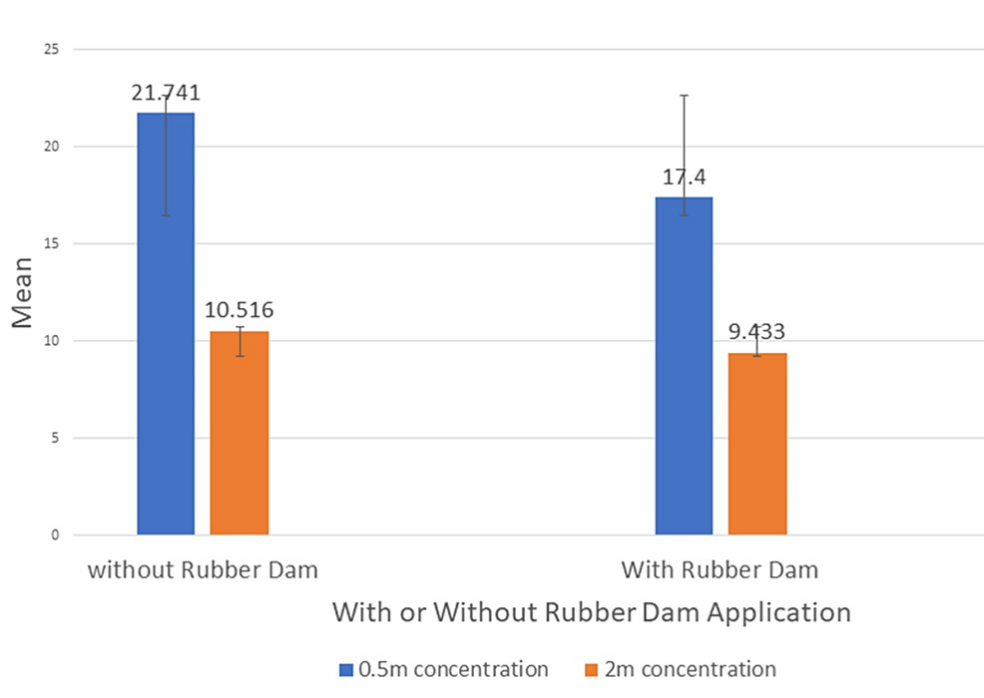
Graph showing intragroup comparison of CFU count between 0.5 m and 2 m distances. CFU - colony forming unit

The mean CFU count at the 0.5 m distance was 21.741±2.081 in the without rubber dam group and 17.400±1.734 in the rubber dam group. The mean CFU count at the 2 m distance was 10.516±1.338 in the without rubber dam group and 9.433±1.612 in the rubber dam group (Figure 5). There was an intragroup reduction in the aerosol count when the distance was increased from 0.5 m to 2 m.

## Discussion

Aerosol particles generated during dental treatment range in size from 1.3 µm to 7.0 µm. Particles smaller than 5.0 µm can enter the airway and penetrate the terminal bronchioles and alveoli of the lungs [5]. Because they are minute, these particles are invisible to the human eye and can remain airborne for an extended period.

Dental aerosols may consist of saliva, nasopharyngeal secretions, gingival crevicular fluid, blood, tooth components, and any substances used during dental procedures, including abrasives for air polishing and air abrasion. During dental procedures, the microbial contamination in airspace can be amplified two to three times in comparison to the levels before and after the appointment [6]. However, it is important to note that the contamination level does not promptly revert to its initial baseline.

Air change per hour (ACH) or air change rate simply refers to the number of times air gets replaced in each room every hour [7]. Dental clinic design standards use ACH for proper ventilation and prevent the accumulation of contaminated aerosol particles in the operatory environment by allowing the circulation of fresh air [8]. Recommended ACH values for dental operatories are 6-10 [9]. High vacuum suctions along with pre-treatment rinse with an antimicrobial agent and replacement of physical barrier after every case are some of the measures of infection control [10].

We conducted a study on aerosolized microorganisms by employing the passive air sampling technique known as “settle plates.” This particular sampling technique has gained significant popularity in the field of dentistry. The primary objective of this method is to quantify the viable microorganisms that have the potential to settle, thrive, and reproduce on a plate [11,12]. Petri dishes with culture media are utilized to capture biological particles from the air over a specific period. After sedimentation, these particles are then incubated, and the findings are quantified in CFUs [13,14]. Aerosols generated while doing the procedure have the potential of cross-contamination even up to 2 m in patients without using a rubber dam [15,16].

However, this study does not cover patients in which rubber dam placement is not possible, and other methods of preventing aerosol generation need to be compared for further evaluation.

## Conclusions

The implementation of rubber dam application in dental facilities offers an effective and practical strategy to minimize the spread of infectious pathogens by reducing aerosol generation. It provides a physical barrier between the patient's oral cavity and the surrounding environment, preventing the transmission of microorganisms during dental procedures. Moreover, it improves visibility, access, patient comfort, and safety, making it a valuable tool in modern dentistry.
